# Using Collisional Electron Spectroscopy to Detect Gas Impurities in an Open Environment: CH_4_-Containing Mixtures

**DOI:** 10.3390/molecules27186066

**Published:** 2022-09-16

**Authors:** Chen Zhou, Jingfeng Yao, Lezhi Zhan, Chengxun Yuan, Anatoly Kudryavtsev, Almaz Saifutdinov, Ying Wang, Zhi Yu, Zhongxiang Zhou

**Affiliations:** 1 School of Physics, Harbin Institute of Technology, Harbin 150001, China; 2 School of Environment, Harbin Institute of Technology, Harbin 150001, China; 3 Department of Chemistry, University College London, London WC1E 6BT, UK; 4 Heilongjiang Provincial Key Laboratory of Plasma Physics and Application Technology, Harbin 150001, China; 5 Physics Department, Saint Petersburg State University, 198504 St. Petersburg, Russia; 6 Department of General Physics, Kazan National Research Technical University Named after A. N. Tupolev, 68 Karl Marx, 420015 St. Kazan, Russia

**Keywords:** non-local plasma, collisional electron spectroscopy, gas impurities detection, fast electron, open environment

## Abstract

The collisional electron spectroscopy method for analyzing and determining gaseous impurities was further developed to realize the operation in an open environment. In addition, the method not only facilitates the registration of the impurity components, but also the reactive radicals generated from the discharge reaction. The sandwich-like discharge structure was used to generate a stable, non-local, negative glow equipotential plasma in an open environment, and the *I*–*V* characteristic curve of the plasma was collected using an additional sensor electrode. The collisional electron spectroscopy was obtained from the first derivative of the probe current *I* with respect to the probe potential *V* by adding a diffusion function to correct it. In addition, our experiment verifies the reliability of the sink theory.

## 1. Introduction

Existing methods to detect chemical components in the gas phase include UV-visible differential optical absorption spectroscopy [1], cavity ring-down spectroscopy [2], cavity-enhanced absorption mass spectrometry [3], gas chromatography and other traditional electron spectroscopy methods [4,5,6]. However, all of these analytical methods are limited due to their high vacuum requirements, difficulties in gas sampling, and the need for delivery from a high-pressure atmosphere to an ionization chamber, which contributes to the large size and complexity of the analytical equipment. To overcome these limitations, the plasma electron spectroscopy (PLES) method was developed to investigate the fundamental plasma process and atomic property [7,8], which provided a modern approach to the registration of gas impurities. Subsequently, Kudryavtsev developed a new method based on plasma electron spectroscopy called collisional electron spectroscopy (CES) [9] for registration of gas impurities in non-vacuum conditions. The method identified gas impurities by selectively recording non-local fast electrons, which were generated by collisions of metastable atoms B^*^ with impurity atoms, named molecules *A.*

(1) $$A+B^{*}\to A^{+}+B+e\left\{E_{p}\right\}$$

(2) $$B^{*}+e\to B+e\left\{E_{m}\right\}$$

As the ionization potential *E_i_* of each type of atom or molecule is a well-known atomic constant, measuring the energy of Penning electrons *E_p_* and using the well-known excitation energy of buffer carrier gas (rare gas) atoms, it is possible to identify the atoms or molecules A of unknown impurities with their ionization potential, $E_{i}=E_{m}-E_{p}$. The method is founded on the observation of the energy spectrum for the fast electrons released during the Penning ionization of impurity atoms or molecules with metastable atoms in the buffer carrier gas. It is noteworthy that the preference for B* particles in reaction (1) is metastable helium (He) that has an excitation energy of 19.8 eV, which is enough to ionize almost any impurity (although not neon).

In contrast to the PLES method, the CES method uses a short DC micro-discharge of negative glow plasma as the most appropriate medium on which to base the non-local formation of the electron distribution function (EDF). The criterion of non-local EDF can be characterized by the form of [10].

(3) $$\lambda_{\varepsilon }>L$$

Here, $\lambda_{\varepsilon }$ is the electron energy relaxation length and *L* is plasma dimension.

In this condition, the EDF is the same at any point of the plasma and does not depend on the local plasma parameters. The non-local negative glow plasma has many advantages, such as the high rate of Penning ionization, high metastable atom density, small plasma volume with low electron temperature, and low operating voltage [11]. This method facilitates the identification of gaseous impurities in the working gas that are in a collisional state. When inequality (3) is satisfied, different groups of electrons behave independently of each other as they do not have time to ‘mix’ due to the collisions during their movement from their place of origin to the boundaries of the plasma volume. More details on the process can be found in [12]. Another advantage of this method is that it can be used at high pressures and improves sensitivity. As a result, the analysis of atoms and molecules of impurities is carried out by registering the electron energy generated by the collision of a metastable atom with an impurity atom or molecule. There are several publications on the CES method for impurity gas detection [11,12,13]. However, these papers only report studies on atoms, inorganic molecules, and metal ions interacting with metastable atoms of the main gas, which could be pure He, He/Ar, He/Kr, He/N_2_, He/O_2_, He/CO_2_, pure Ar, or Ar/Pt. It is worth noting that these experiments were performed in enclosed environments under low pressure. In addition, almost all of the impurity gases detected using the CES method are atoms and inorganic molecules, which greatly improves the accuracy of the diagnostics due to their stable chemical behavior.

Although the chemical behavior of organic substances is unstable and has complex chemical reactions, they play an extremely important role in people’s lives. Methane (CH_4_) is one of the most common organic substances and alternative resources to produce chemical products and liquid fuels compared to oil that contributes to global warming. It has abundant reserves in the form of natural gas. However, CH_4_ leaks are inevitable during the extraction, transport, and utilization of natural gas. The timely detection and extraction of methane can greatly reduce the harmful effects of its leakage. Therefore, it is important to find a portable, low-cost, and highly sensitive detection method. Stepaniuk et al. [14] reported the detection of CH_4_; however, they found some characteristic electronic peaks have not been clearly explained. In addition, the experiment was performed with plasma generated by a pulsed glow discharge and conventional Langmuir probe located in a positive column with the PLES method and gas chromatography. It is well known that most of the electrons in the positive column region of plasma have high electron temperatures (between several and a dozen eV), and the electron temperature of the fast electrons being ionized in this experiment is also in this range, making the analysis of the experimental results much less accurate. These things considered, the conventional Langmuir probe has a small radius size making its diagnostic sensitivity relatively low. In addition, the experiment was carried out in a closed glass tube with a vacuum pump to adjust the pressure; thus, the experimental conditions were extreme. Previously, our group studied CH_4_-containing mixture purities; however, the experimental environment was also based on a vacuum environment that could not satisfy the practical requirements [15].

In this study, a sandwich-like discharge structure is used to generate a stable, non-local, negative glow equipotential plasma in an open environment. At the same time, an additional electrode is introduced as a sensor to collect the *I*–*V* characteristic curve of the plasma. For CH_4_-containing mixtures (99.85% He + 0.1% CH_4_ + 0.05% O_2_), we obtained the EDF with characteristic electron peaks and report it here, using the first derivative of the *I*–*V* characteristic curve with a correction factor in an open environment. Different correction factors are used to make the characteristic electron peaks more visible.

## 2. Experimental Setup

**Figure 1:**
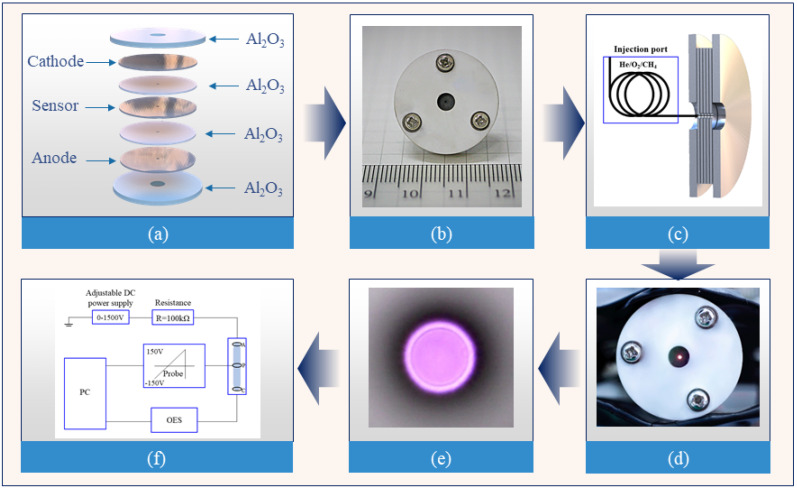
Sandwich-like construction: (**a**) schematic; (**b**) photograph; (**c**) schematic of the profile; (**d**) discharge photograph; and (**e**) photograph of the plasma. (**f**) Block diagram of the experimental setup.

For the experiment, we worked with a sandwich-like micro-discharge device with an additional sensor that can generate a diagnosable and stable plasma source in an open environment. The probe diagnostic method is considered to be the one of the most accurate methods for the determination of plasma characteristics. However, it is impossible to introduce a conventional Langmuir probe into the plasma in small regions. Therefore, we employed an additional wall electrode as a sensor, which was located between the cathode and anode to register the EDF with fast electrons produced in the Penning ionization reaction across a high-pressure range (atmospheric pressure). As a result, the traditional Langmuir probe with a small radius *a* (a<λ, λ is the mean free path of the electrons) was placed in the plasma to measure the EDF, in contradistinction to the non-local plasma where the EDF can be recorded by an additional sensor electrode located at the plasma boundary. Sensor electrodes do not cause plasma distortion. The relatively large acquisition area leads to increased sensitivity of the sensor. The increased sensor surface (equivalent to the anode surface) was used to collect a large number of Penning electrons and the extreme values in the electron energy spectrum were easily obtained at medium voltage and low discharge currents. The schematic of the plasma generation device is shown in Figure 1a [16]. It contains three pieces of molybdenum and two pieces of ceramic (Al_2_O_3_). The molybdenum sheets were employed as the anode (0.2 mm-thick), cathode (0.2 mm-thick), and sensor (0.2 mm-thick), and the ceramic (0.15 mm-thick) acted as a dielectric. In the device, a hole with a radius of 0.05 mm was punched with a laser (Figure 1b); thus, the wall probe had a radius of 0.05 mm. For elastic electron–atom collisions in noble gases, the criterion for EDF nonlocality was met for p×L<10 Torr cm, where *p* is the gas pressure and *L* is a characteristic plasma dimension. As a result, *L* should be of the order of a few cm for 1 Torr pressure and 0.1 mm for atmospheric-pressure noble gases [17,18]. In order to achieve a stable discharge, helium and its mixtures were pumped into the middle hole (Figure 1c). A block diagram of the experimental setup is shown in Figure 1f. For this experiment, a 0–1500 V adjustable DC supply with a readable current was selected, with the use of a 100 kΩ resistance to secure the circuit.

The CES method involved the use of a probe measurement technique. Over the past few decades, a large amount of related work has been published and widely reviewed [19,20,21]. Digital to analog converters are combined to provide very fast data acquisition, and a commercially available Langmuir probe system is used in experiments, which includes a compensation probe for high voltage discharge. Nevertheless, as plasma processing becomes more complex, more sensitive methods are needed to obtain the EDF. Specifically, a method of testing functions is proposed in [22]. The hardware function needs to be considered when registering the fast part. Furthermore, in hydrocarbon-containing gases, polymer films are formed which distorts the signal [23]. 

In this study, the Langmuir single-probe tool was used for data acquisition, where the voltage scale was −150 to +150 V, and the scan step value was 0.2 V. In previous studies [16], the *I*–*V* characteristics obtained from experimental results showed an increasing trend when the discharge current was small, and a decreasing trend when the discharge current was large. During the transition, the discharge region changes from an anomalous glow discharge region to a normal discharge region. It is difficult to obtain current parameters at quite high voltages using Langmuir probes due to the limited measurement range of the probe system. Considering the above factors, we selected the anomalous glow discharge region for our experimental study. The discharge voltage utilized in this experiment was 490 V, the corresponding current was 10.7 mA, and the gas flow rate was 5 mL/min. The concentration of pure helium was 99.999%, and the gas mixture was 99.85% He + 0.1% CH_4_ + 0.05% O_2_ pre-mixed standard gas. The gas mixtures were produced by a commercial gas company and were operated via a flowmeter. Figure 1d shows a photograph of the plasma generated from the discharge of the experimental set-up. The bright spot in the middle is the plasma generated from the ionization of the detected gas. Figure 1e shows a magnified view of the bright spot in Figure 1d.

**Figure 2:**
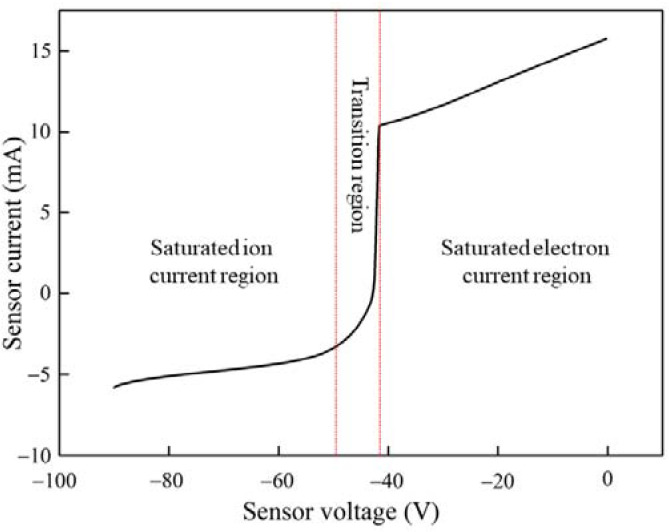
*I*–*V* characteristic of wall probe at atmospheric pressure with a flow rate of 5 mL/min for He and the discharge current of 10.7 mA, which has a consistent *I*–*V* characteristic obtained by traditional Langmuir probe with the saturated electron and saturated ion current region and the transition region.

Figure 2 shows the *I–V* characteristic generated by the pure He discharge, which is similar to that obtained with the conventional Langmuir probe [24]. It involves the saturated electron and ion current region and the transition region. The electron temperature is determined with the well-known formula that employs the linear part of the transition region [20,21]:

(4) $$T_{e}=\left|\frac{e}{k}\frac{\Delta U}{\Delta \left(\ln i^{\prime }\right)}\right|=\left|\frac{e}{k}\frac{\Delta U}{\Delta \left(\ln i^{\prime \prime }\right)}\right|$$

where $\Delta \left(\ln i^{\prime }\right)$ and $\Delta \left(\ln i^{\prime \prime }\right)$ represent the logarithmic change in the derivative of the current entering the sensor. When the change in the potential of the sensor is Δ*U*, *e* is the fundamental electric charge, *k* is the Boltzmann constant, and $i^{\prime }$ and $i^{\prime \prime }$ represent the first and second derivative of the probe current, respectively.

The result shows that the electron temperature value was 0.62 eV, which is typical for a negative glow plasma. It should be noted that equation (4) was established with the Maxwell distribution function for estimating the temperature of the main group of electrons, which is true for a negative glow plasma, since the distribution function of electrons in a negative glow plasma is strongly affected by the interelectronic collisions that lead to its Maxwellization. In addition, the experiments were carried out at high pressure, and for correct interpretation it is necessary to consider the collisions in the near-probe layer and the electron sink. Failure to take these factors into account leads to an overestimation of the measured temperature. Consequently, the plasma still retains the property of a negative glow plasma (*T_e_* < 1 eV), which does not affect the detection of fast electrons (>1 eV) by the probe.

## 3. Results and Discussion

Probe diagnostics at a high pressure represent a common research topic in plasma diagnostics. In 1994, a kinetic theory for the electron probe current was presented by Arslanbekov et al. [25]. Their result allows us to infer the EDF from the measured electron probe currents and their derivatives over a wide range of pressures. The effect of the electron sink connected to the probe is reduced electron density near the probe due to electron absorption (recombination) by the probe surface. Distortion of the EDF occurs due to the finiteness of the rate of diffusion; furthermore, replenishment of the electrons in the region is depleted due to their flow to the probe. Demidov et al. used kinetic probe theory to study and analyze how the Langmuir probe theory is modified in the case of electron/ion/atom collision in the sheath. More details on the process can be found in [26]. As a result, the current obtained by the probe is less than the actual current, which makes it inappropriate to obtain the EDF from the Druyvesteyn Equation (5) of the collision-free model [27].

(5) $$I_{e}(V)=\frac{2\pi N_{e}S}{m^{2}}\int_{eV}^{\infty }f\left(\varepsilon \right)\left(\varepsilon -eV\right)d\varepsilon$$

Here, *I_e_* is the electron current collected by the probe, $N_{e}$ is the electron density, *S* is the probe surface area, *m* is the mass of the electron, *V* is the (negative) probe potential, $f\left(\varepsilon \right)$ is the EDF, and *ε* is the electron energy.

In this paper, it is assumed that for the distances from the probe surface starting at the infinity to the mean free path of electrons, λ, electrons flow to the probe due to diffusion and from distance λ to the probe surface they go to the probe freely without collisions. It is also assumed that the mean-free-path of electrons and the probe radius are much greater than *r_D_*. The theory based on the above assumption is named sink theory [26,28]. Here, Swift first added a correction term to the Druyvesteyn Equation (5) of the collision-free model [29], which is expressed as

(6) $$\delta \left(\varepsilon \right)=\frac{3a^{2}}{4\lambda \left(\varepsilon \right)\left(a+\lambda \left(\varepsilon \right)\right)}$$

Here, $\delta \left(\lambda \right)$ is the sink parameter, $\lambda \left(\varepsilon \right)$ is the mean free path of the electron, and *a* is the radius of probe.

Then, we can obtain the modified Equation (7) based on the above assumption.

(7) $$I_{e}\left(V\right)=\frac{2\pi N_{e}eS}{m^{2}}\int_{eV}^{\infty }\frac{f\left(\varepsilon \right)\left(\varepsilon -eV\right)d\varepsilon }{1+\delta \left(\varepsilon \right)\left(1-\frac{eV}{\varepsilon }\right)}$$

From Equation (7), specifically the differentiation over the probe potential, one obtains [30]

(8) $$f\left(\varepsilon \right)=\frac{\delta \left(eV\right)m^{2}}{2\pi N_{e}SVe^{3}}\times \frac{dI}{dV^{\prime }}$$

which is an analogue of the Druyvesteyn equation for a collisional plasma, and $V^{\prime }$ is the first derivative of the (negative) probe potential.

Note that

(9) $$\lambda \left(\varepsilon \right)\frac{1}{n_{g}\sigma }$$

Here, *n_g_* is the density of neutral gas, and σ is the collision cross section. Therefore, by assigning $\lambda \left(\varepsilon \right)$ and *a* to Equation (6), the $\delta \left(\varepsilon \right)$ is obtained.

**Figure 3:**
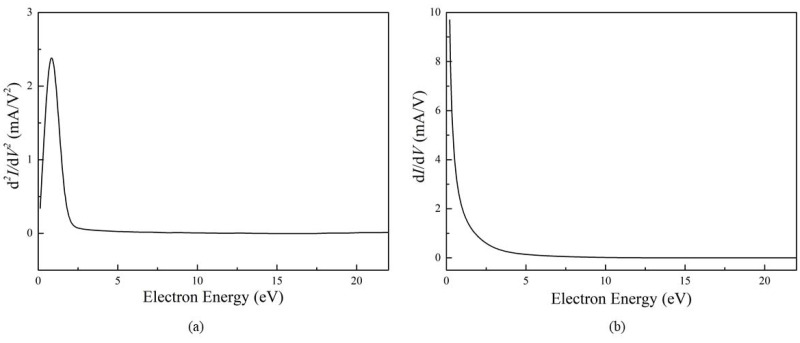
The second (**a**) and first (**b**) derivative of the probe current with respect to the potential for helium at atmospheric pressure with a flow rate of 5 mL/min and the discharge current of 10.7 mA.

Figure 3a shows the second derivative of the *I*–*V* characteristic by the probe in Figure 2. Because the experiment was carried out at a high pressure, there was collision behavior between the electrons. As a result, the EDF could not be determined by the Druyvesteyn equation and the second derivative of the *I*–*V* characteristic. Therefore, the characteristic electron peak is not visible in the EDF. Figure 3b shows the first derivative of the *I*–*V* characteristic by the probe in Figure 2, and the corresponding characteristic electron peak was also not obtained.

**Figure 4:**
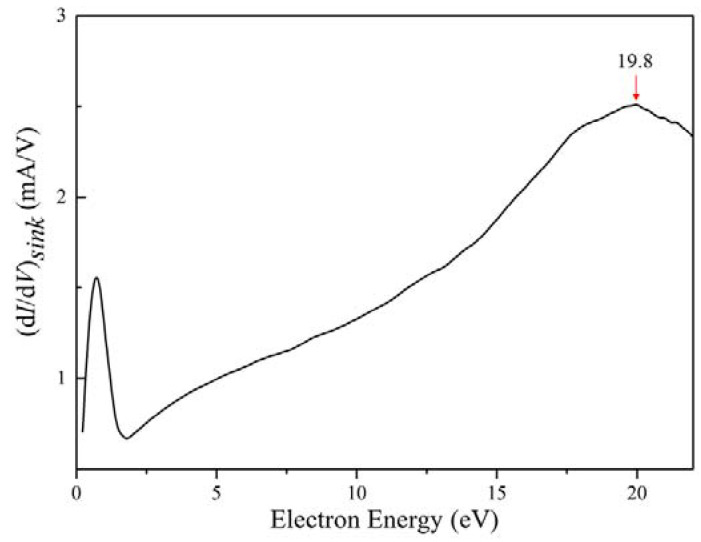
The first derivative modified by sink parameters for helium at atmospheric pressure with a flow rate of 5 mL/min and the discharge current of 10.7 mA, where 19.8 eV is the characteristic electron peak of He.

Figure 4 shows the results obtained after the modification of the first derivative of the *I*–*V* characteristic curve using the sink parameter, (d*I*/d*V*)*_sink_*. A characteristic electron peak is evident at approximately 20 eV, which is due to the electron peak generated by the collision of electrons with metastable He:

(10) $$\mathrm{He}(2^{3}S_{1})+e\to \mathrm{He}\;+e\left\{19.8\;\mathrm{eV}\right\}$$

Here, the characteristic electron peak in the EDF broadens due to the increasing role of elastic collisions with increasing pressure compared to diffusion.

**Figure 5:**
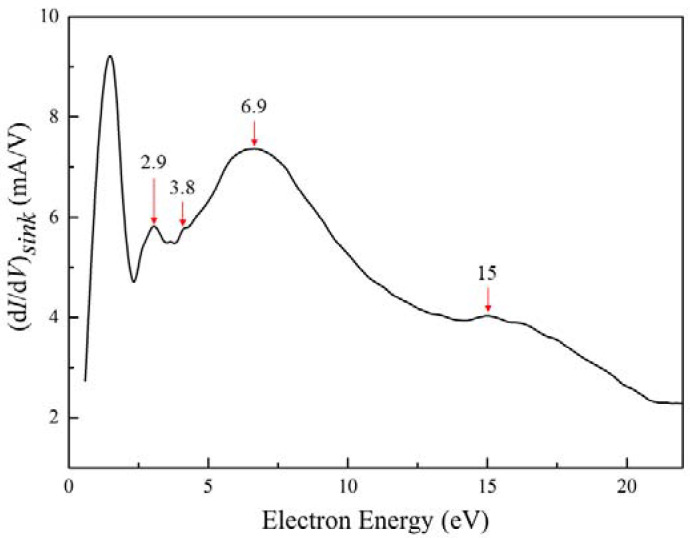
The first derivative modified by sink parameters for CH_4_–containing mixtures at atmospheric pressure with a flow rate of 5 mL/min and the discharge current of 10.7 mA, where 2.9, 3.8, 6.9 and 15 eV are the characteristic electronic peaks of O, CH, CH_4_ and He, respectively.

The existence of analytes will influence the plasma parameters, especially by causing changes of the plasma potential, which will result in a shift of the *I*–*V* characteristic curve along the voltage axis. A helium peak appears in each of the “raw” CES spectra caused by the Penning ionization due to the collision of two helium metastable atoms, which makes the absolute calibration of the voltage axis as possible. Based on the above method, we further report the CES of CH_4_–containing mixtures. The experimental result is shown in Figure 5. There are four characteristic fast–electron peaks in the electron energy spectrum: at 2.9, 3.8, 6.9, and 15 eV. The peak at 15 eV is produced by the collision of two metastable helium atoms, as shown below.

(11) $$\mathrm{He}(2^{3}S_{1})+\;\mathrm{He}(2^{3}S_{1})\to {\mathrm{He}+\mathrm{He}}^{+}+e\left\{15\;\mathrm{eV}\right\}$$

The existence of analytes influences the plasma parameters, specifically by causing changes of the plasma potential, which result in a shift of the *I*–*V* characteristic curve along the voltage axis. Therefore, it is reasonable that the characteristic electron peaks obtained from the collisions are smaller than the calculated values. For the peak of 6.9 eV, the following response will appear: ($E_{{\mathrm{CH}}_{4}}=12.9\;\mathrm{eV}$) [14].

(12) $$\mathrm{He}(2^{3}S_{1})+{\;\mathrm{CH}}_{4}\to {\mathrm{He}+\mathrm{CH}}_{4}^{+}+e\left\{6.9\;\mathrm{eV}\right\}$$

This characteristic peak is not sharp, which is due to the fact that the speed of the fast electron was much greater than the speed of the bulk electron, so the Coulomb force of the charged particle is not ignored. With the assumption of an electrically neutral plasma, the fact that fast electrons are slowed down by ions was taken into account and interpreted as an influence of ambipolar diffusion. It is worth noting that this characteristic peak was also obtained by Stepaniuk et al. using the second derivative method, and the result was verified by performing gas chromatography [14].

Due to the collisions of metastable helium with each other, high-energy electrons (e^*^) have an “electron activation” effect on CH_4_ and O_2_, which consists of the following dissociation reactions of CH_4_ and O_2_ [31]:

(13) $${\mathrm{CH}}_{4}+e^{*}\to {\mathrm{CH}}_{3}+H+e$$

(14) $${\mathrm{CH}}_{3}+e^{*}\to {\mathrm{CH}}_{2}+H+e$$

(15) $${\mathrm{CH}}_{2}+e^{*}\to \mathrm{CH}+H+e$$

(16) $$\mathrm{CH}+e^{*}\to C+H+e$$

(17) $${\mathrm{CH}}_{4}+e^{*}\to {\mathrm{CH}}_{2}+2H\left(H_{2}\right)+e$$

(18) $${\mathrm{CH}}_{4}+e^{*}\to \mathrm{CH}+3H\left(H_{2}+H\right)+e$$

(19) $${\mathrm{CH}}_{4}+e^{*}\to C+4H\left({2H}_{2}\right)+e$$

(20) $$O_{2}+e^{*}\to 2O+e$$

From the reactions listed above, it is clear that high-energy electrons (19.8 eV) generated by collisions between metastable helium will activate methane and oxygen to cause their dissociation. This explains why the probe did not detect high-energy electrons of this kind (19.8 eV). As a result, the characteristic peak at 3.8 eV is derived from the following reactions: ($E_{{\mathrm{CH}}_{2}}=16\;\mathrm{eV}$, $E_{\mathrm{CH}}=16\;\mathrm{eV}$) [32,33].

(21) $$\mathrm{He}(2^{3}S_{1})+{\;\mathrm{CH}}_{2}\to {\mathrm{He}+H+\mathrm{CH}}^{+}+e\left\{3.8\;\mathrm{eV}\right\}$$

(22) $$\mathrm{He}(2^{3}S_{1})+\;\mathrm{CH}\to {\mathrm{He}+H+C}^{+}+e\left\{3.8\;\mathrm{eV}\right\}$$

Related studies have shown that the above 3.8 eV characteristic electron peak is more likely to be the characteristic electron peak of CH [34]. For the production of CH species, studies using the isotope–labeled method offer two pathways. One reason is that CH_4_ can directly produce CH radical species when subjected to high–energy electron activation. A single inelastic collision of an electron with CH_4_ can dissociate multiple C–H bonds. The plasma process formed during the discharge of methane is mainly a free–radical process, and the total electron energy required to completely break the C–H bond contained in the methane gas molecule is of the order of tens of eV [35]. In fact, it is unlikely to form such high–energy electrons. However, to break each C–H bond in turn requires an energy of only about 4.5 eV [36], and the probability of forming such energetic electrons is quite high. For another reason, when the plasma density is high, CH_4_ can also generate C radical species by means of continuous dehydrogenation (Equations (17)–(19)). The existence of the above process is possible due to the high energy of the electrons produced by the mutual collisional ionization between metastable helium atoms and its high density in the plasma, since the experiment was carried out in atmospheric pressure. Another possibility is that CH_2_ and CH_3_ are less stable than CH. Under the collision of high-energy electrons, it is very easy to decompose into CH.

The characteristic peak at 2.9 eV is derived from the following reaction ($E_{O{(}^{2}D)}=16.9\;\mathrm{eV}$) [37].

(23) $$\mathrm{He}(2^{3}S_{1})+\;O{(}^{2}D)\to {\mathrm{He}+O}^{+}+e\left\{2.9\;\mathrm{eV}\right\}$$

Here, O is produced from the collisional dissociation of oxygen by electrons (Equation (22)).

**Figure 6:**
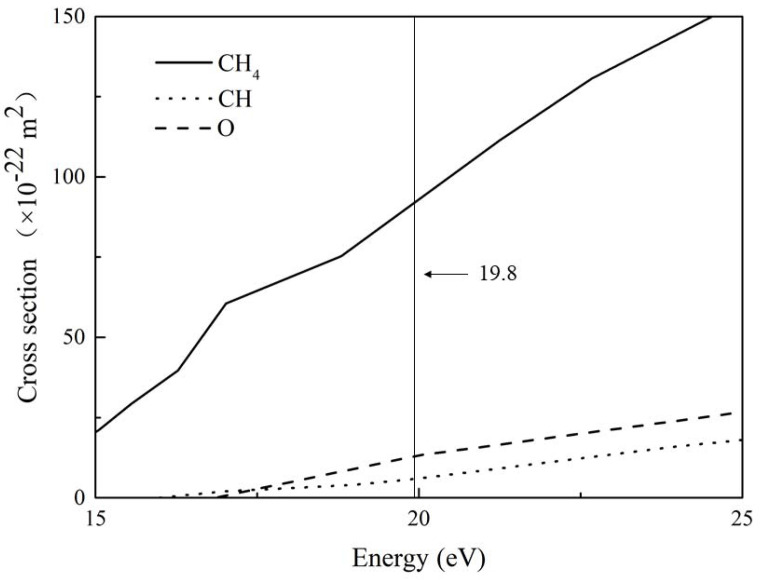
Partial cross section data of CH_4_ (solid line), CH (dot line), and O (dash line) obtained from the Plasma Data Exchange Project [33,37,38].

Figure 6 shows the collision cross section of O, CH, and CH_4_ at 19.8 eV. Here, the data are selected from the database of the Plasma Data Exchange Project [33,37,38]. This figure shows that the collision cross section of CH_4_ is much larger than that of CH and O at an electron energy of 19.8 eV, and the collision cross section of CH is slightly larger than that of O. This means that CH_4_ has a much higher collision probability than the CH and O under the same conditions, which corresponds to the height of the characteristic peak of fast electrons, as shown in Figure 5. This verifies the accuracy of our experimental results.

## 4. Conclusions

Detection and analyses of methane-containing gas mixtures in the open environment were successfully achieved with additional sensor electrodes. This experiment also verifies that the method of obtaining the EDF by introducing sink theory to correct the first derivative of the *I*–*V* characteristic at high pressures is valid. For the mixture containing CH_4_, fast electron characteristic peaks were obtained at 2.9, 3.8, 6.9, and 15 eV, which correspond to O, CH, CH_4_, and He ($2^{1}S_{0}$), respectively. In addition, hydrocarbon impurities, which form different types of radicals, such as CH, can also be registered successfully using the CES method. The advantages of our method are the portability of the equipment, the low cost, and the capability to perform multiple chemical analyses of atomic, molecular, and ionic fragments with CES-based qualitative analyses. The detection system has the potential to become a fast-response system for gas impurity analysis, thus enabling online detection.

## Data Availability

The data that supports the findings of this study are available within the article.
